# Model-Based Investigations of Different Vector-Related Intervention Strategies to Eliminate Visceral Leishmaniasis on the Indian Subcontinent

**DOI:** 10.1371/journal.pntd.0002810

**Published:** 2014-04-24

**Authors:** Anette Stauch, Hans-Peter Duerr, Albert Picado, Bart Ostyn, Shyam Sundar, Suman Rijal, Marleen Boelaert, Jean-Claude Dujardin, Martin Eichner

**Affiliations:** 1 Department of Clinical Epidemiology and Applied Biometry, University of Tübingen, Tübingen, Germany; 2 Numerus Limited, Tübingen, Germany; 3 Barcelona Centre for International Health Research (CRESIB, Hospital Clínic-Universitat de Barcelona), Barcelona, Spain; 4 Department of Public Health, Institute of Tropical Medicine, Antwerp, Belgium; 5 Department of Medicine, Institute of Medical Sciences, Banaras Hindu University, Varanasi, India; 6 Department of Medicine, BP Koirala Institute of Health Sciences, Dharan, Nepal; 7 Department of Biomedical Sciences, Institute of Tropical Medicine, Antwerp, Belgium; 8 Laboratory of Microbiology, Parasitology and Hygiene, University of Antwerp, Antwerp, Belgium; Oswaldo Cruz Foundation, Brazil

## Abstract

The elimination of infectious diseases requires reducing transmission below a certain threshold. The Visceral Leishmaniasis (VL) Elimination Initiative in Southeast Asia aims to reduce the annual VL incidence rate below 1 case per 10,000 inhabitants in endemic areas by 2015 via a combination of case management and vector control. Using a previously developed VL transmission model, we investigated transmission thresholds dependent on measures reducing the sand fly density either by killing sand flies (e.g., indoor residual spraying and long-lasting insecticidal nets) or by destroying breeding sites (e.g., environmental management).

Model simulations suggest that elimination of VL is possible if the sand fly density can be reduced by 67% through killing sand flies, or if the number of breeding sites can be reduced by more than 79% through measures of environmental management.

These results were compared to data from two recent cluster randomised controlled trials conducted in India, Nepal and Bangladesh showing a 72% reduction in sand fly density after indoor residual spraying, a 44% and 25% reduction through the use of long-lasting insecticidal nets and a 42% reduction after environmental management.

Based on model predictions, we identified the parameters within the transmission cycle of VL that predominantly determine the prospects of intervention success. We suggest further research to refine model-based predictions into the elimination of VL.

## Author Summary

Visceral leishmaniasis is suspected to be the second largest parasitic killer in the world after malaria. On the Indian subcontinent, the vector-borne disease is caused by the protozoan flagellate *Leishmania donovani* and transmitted by the sand fly *Phlebotomus argentipes*. The regional elimination programme has suggested as a target line to reduce the annual incidence below 1 case in 10,000 by 2015. Using a previously developed mathematical model, we investigated to what extent the sand fly population must be controlled to achieve elimination. These calculated thresholds were compared to data from two recent trials conducted in India, Nepal and Bangladesh to evaluate the efficacy of different vector control measures. Our results indicate that elimination should be feasible because the evaluated effect of indoor residual spraying exceeds the threshold. However, emerging insecticide resistance may compromise the effectiveness of this measure. The observed effects of long lasting insecticidal nets and environmental management do not seem to be sufficient to reach either threshold. Integrated vector management based on indoor residual spraying combined with long lasting insecticidal nets and more effective environmental management may allow overcoming the limitations of the current vector control methods and should also prevent re-emergence of the infection after local extinction.

## Introduction

Visceral leishmaniasis (VL), also known as Kala-azar, is a vector-borne parasitic disease that almost always ends in death if untreated [1]. Infection with the parasite, *Leishmania chagasi/infantum* in the New or parts of the Old World or *Leishmania donovani* in other regions of the Old World, occurs through the bite of an infected sand fly and leads to a spectrum of outcomes ranging from asymptomatic infection to active disease. In the latter case, progressive clinical manifestations, including prolonged fever, weight loss, hepatosplenomegaly and anaemia, ultimately result in suppressed immune responses and death [2]. Most infections do not lead to symptoms but proceed asymptomatically [3], [4]. VL is endemic in 79 countries and causes between 0.2 to 0.4 million cases and 20,000 to 40,000 deaths per year [5]. Clinically, VL cases are often not detected, not reported or erroneously diagnosed as malaria [6], [7]. VL epidemics are geographically clustered and usually occur in remote locations affecting primarily the poorest of the poor [8]. In the Global Burden of Disease study, VL is ranked as the second largest parasitic killer in the world after malaria [9]. On the Indian subcontinent, the majority of cases are reported in northeastern India, mainly in the state of Bihar, followed by neighbouring regions in Bangladesh and the southeastern Terai region of Nepal. There, VL is caused by *L. donovani* and transmitted by the female sand fly *Phlebotomus argentipes* in an anthroponotic cycle [10].

The national malaria eradication campaigns from 1953 to 1964 nearly achieved elimination of VL throughout the South Asian subcontinent as a side-effect of the DDT-spraying in houses [11]. Molecular data show remarkable homogeneity in current strains from this region, suggesting that the *Leishmania* parasites had previously passed through a genetic bottleneck [12]–[14], an occurrence that can be attributed to the near elimination of *L. donovani* from the Indian subcontinent during that era. Starting in 1956, no further cases of VL were reported in the region. After termination of the malaria eradication campaign, it was thought that VL had been eliminated from Bihar and the presence of patients with chronic disease or post-kala-azar dermal leishmaniasis (PKDL) was ignored. Soon, the vector density rebounded, new cases re-emerged in Bihar and approximately 4500 deaths were reported during a 1977 epidemic [11], [15]–[18]. This first epidemic was followed by two other major VL epidemics, reflecting the experience that VL occurs in cycles of 10 to 20 years [19].

In May 2005, the governments of India, Nepal and Bangladesh signed a Memorandum of Understanding to eliminate VL. The objective of this programme is to reduce the incidence of VL below 1 case per 10,000 inhabitants per year by 2015 in endemic areas [20]. Current VL elimination strategies involve early case detection, effective treatment and vector control by indoor residual spraying (IRS). Insecticide-treated nets and interventions reducing the breeding capacity of *P. argentipes* have been proposed as alternatives or complements to IRS [15]. However, scientific evidence on the impact of the different vector control strategies is still weak [21], [22].

On the Indian subcontinent, only two recent cluster randomised trials have studied the efficacy of different vector control measures using indoor *P. argentipes* density as an outcome variable [21]. Joshi *et al.* [23] conducted a cluster randomised controlled trial in India, Nepal and Bangladesh from 2006 to 2007 to evaluate the impact of IRS, long lasting insecticidal nets (LLIN) and environmental management (EVM) on sand fly density. In the IRS clusters (n = 24), walls of houses and cattle sheds were sprayed using three different insecticides: DDT in India, deltamethrin in Bangladesh and alpha-cypermethrin in Nepal. In the LLIN clusters (n = 24), deltamethrin-treated bed nets were distributed to all households. Finally, in the EVM clusters (n = 24), cracks and crevices in houses and cattle sheds were plastered with a lime/mud mixture (India and Nepal) or with mud only (Bangladesh) to destroy the sand flies' breeding sites. The data showed a 72% reduction in sand fly density for IRS, a 44% reduction for LLIN and a 42% reduction for EVM after 5 months, compared to control clusters (n = 24).

Picado *et al.* [24] reported the results of a cluster-randomised trial (KALANET project) evaluating the impact of insecticide-treated nets on indoor *P. argentipes* density in India and Nepal. After 12 months of LLIN use, houses in intervention clusters (n = 6) had a 25% lower *P. argentipes* density compared to controls (n = 6 clusters). However, the trial showed that this reduction had only a limited impact on *L. donovani* transmission; 24 months post-intervention, differences in the risk of infection and disease between intervention and control clusters were not statistically significant [25]. To our knowledge, the KALANET project is the only randomised cluster trial evaluating the impact of vector control measures (e.g., LLIN) on clinical endpoints (e.g., *L. donovani* infection and VL cases).

The objective of our study was to investigate the capability of different vector control strategies to eliminate VL on the Indian subcontinent. The impact of interventions reducing the vector's life expectancy (i.e., IRS, LLIN) or breeding capacity (i.e., EVM) were evaluated using a previously published mathematical model on the transmission dynamics of VL [26]. We calculated the basic reproduction number (*R_0_*) and determined the sand fly density for which the elimination threshold, given by an effective reproduction number *R_e_* = 1, is reached. In these calculations, we also considered uncertainties in parameter estimation by varying parameter values within the limits of their joint confidence interval.

## Methods

### Model

We used a previously published VL model [26] that has been used to investigate emerging resistance against antimonial treatment [27]. A system of ordinary differential equations was developed to model the transmission dynamics of *L. donovani* between sand flies and humans on the Indian subcontinent. Model compartments represent the number of sand flies and humans, distinguished according to their diagnostic states. Human hosts are further distinguished by disease and treatment status. Under the assumption of homogeneous spread of *L. donovani* infection, average prevalences taken from data of the KALANET project were used to calibrate the model's equilibrium. The KALANET project was a community intervention trial in India and Nepal conducted between 2006 and 2008 in high transmission clusters with an average VL incidence rate of 2.8/1000 person years (95% CI, 2.1–3.5/1000 person years) [4]. In addition, required data were either taken from the literature or, if published data were unavailable, obtained through expert opinion. In the previously published VL model durations taken from literature to calculate the sojourn time of humans and flies in specific stages are given as duration regardless of mortality, i.e., conditional on surviving whereas in the current approach mortality was considered when calculating sojourn times based on published durations. All parameter values are provided in Table 1 and all parameter descriptions and references are provided in Tables S3 to S6 in the Supplement. Known temporal or spatial heterogeneities were not considered within this modelling approach. Therefore, model results should be interpreted as the portrait of a situation within a highly endemic cluster. Further model details are provided in the Supplement, together with an expression for the effective reproduction number *R_e_*.

**Table 1:** Ranges, means and medians of randomly generated parameter combinations.

	Description	Model Value^A^	Proposed^B^	Accepted^C^^,^ D	
			Range	Range	Mean (Median)
*N_F_*	Breeding site capacity	7,344	4,000–12,000	4,183–11,774	8,047 (8,114)
*μ_F_*	Mortality rate of sand flies*	0.071	0.058–0.082	0.059–0.080	0.070 (0.070)
*σ_F_*	Rate determining the sojourn time of sand flies in the latent stage *E_F_*	0.13	0.05–0.25	0.06–0.24	0.14 (0.14)
*β*	Rate determining the feeding cycle duration	0.178	0.100–0.360	0.134–0.355	0.254 (0.248)
*μ_H_*	Mortality rate of humans	6.85 · 10^−5^	3.30 · 10^−5^–1.40 · 10^−4^	3.33 · 10^−5^–7.80 · 10^−5^	5.08 · 10^−5^ (4.85 · 10^−5^)
*μ_K_*	Excess mortality rate caused by VL	0.0067	0.0001–0.0750	0.0011–0.0696	0.0294 (0.0270)
*p_H_*	Probability that a human becomes infected after being the blood meal of an infected sand fly	1.00	0.38–1.00	0.40–1.00	0.74 (0.74)
*p_F2_*	Probability that a susceptible fly becomes infected when feeding on a DAT-positive but asymptomatic human host#	0.0417	0.0140–0.0720	0.0141–0.0599	0.0349 (0.0341)
*p_F3_*	Probability that a susceptible fly becomes infected when feeding on a symptomatic human host	1.00	0.00–1.00	0.01–1.00	0.48 (0.49)
*f_HS_*	Fraction of DAT-positive but asymptomatic human hosts who develop VL	0.0028	0.0010–0.0100	0.0012–0.0088	0.0050 (0.0051)
*f_HL_*	Fraction of DAT-positive but asymptomatic human hosts who will later develop PKDL	0.00010	0.00000–0.00090	0.00000–0.00086	0.00036 (0.00034)
*γ_HP_*	Rate determining the sojourn time in the early asymptomatic stage *I_HP_*	0.017	0.013–0.026	0.014–0.024	0.019 (0.019)
*γ_HD_*	Rate determining the sojourn time in the early asymptomatic stage *I_HD_*	0.083	0.050–0.140	0.070–0.124	0.097 (0.097)
*γ_HS_*	Rate determining the duration between diagnosis of VL and onset of treatment	0.993	0.010–1.000	0.016–0.997	0.489 (0.469)
*ρ_HD_*	Rate determining the period of DAT-positivity in state *R_HD_*	0.013	0.009–0.020	0.011–0.019	0.016 (0.015)
*ρ_HC_*	Rate determining the period of LST-positivity in state *R_HC_* §	0.00324	0.00250–0.00470	0.00271–0.00468	0.00379 (0.00381)
*δ_HL_*	Rate determining the sojourn time in stage *R_HL_*	0.0015	0.0000–1.0000	0.0049–1.0000	0.5054 (0.4718)
*τ_1_*	Rate determining the sojourn time under first-line VL treatment	0.027	0.000–0.170	0.001–0.158	0.069 (0.068)
*τ_2_*	Rate determining the sojourn time under second-line VL treatment	0.027	0.000–0.170	0.000–0.164	0.076 (0.071)
*τ_3_*	Rate determining the sojourn time under PKDL treatment	0.005	0.000–1.000	0.004–1.000	0.496 (0.521)
*f_1_*	Fraction of VL patients who are not killed by the treatment and do not respond to VL first-line treatment	0.05	0.00–1.00	0.00–1.00	0.48 (0.46)
*f_2_*	Fraction of VL patients who are not killed by the treatment and appear to recover under VL treatment but will develop PKDL	0.03	0.00–0.70	0.00–0.67	0.26 (0.25)
*f_T_*	Fraction of VL patients who die because of treatment	0.05	0.00–1.00	0.00–1.00	0.36 (0.30)

*All rates are specified per day;

# DAT = direct agglutination test;

§ LST = leishmanin skin test;

A : Estimates as in [26] and documented in the Methods section.

B : Ranges of uniform distributions used to randomly generating parameter combinations.

C : Parameter combinations fulfilling the likelihood criterion as documented in the Methods section.

D : For corresponding values of *R_e_* see Figure 2A at 12.5% sand fly reduction.

### Thresholds

*R_0_* summarises how many infections are caused on average by a single infected individual in a non-immune population without intervention. The corresponding number that considers intervention (e.g., vector control, vaccination) is called the effective reproduction number *R_e_*. Elimination of infections in the human population requires *R_e_*<1; i.e., infected humans have to produce on average less than one new infection in humans, leading to extinction of the infection [28], [29]. Depending on the sand fly density, thresholds below which *L. donovani* infections in the human population cannot persist (*R_e_*<1) can be identified. To further investigate this, we modified the model parameters in two steps:

**(a) Screening.** We translated uncertainties in parameter estimation into uncertainties in *R_0_* by first randomly sampling 2,400,000 random values for 23 model parameters from uniform distributions (ranges are shown in Table 1) and then calculated the likelihood of the observations given these values by running the deterministic model until an equilibrium was reached (see [26]). Parameter sets with a log likelihood that deviates by less than 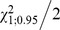 from the maximum likelihood lie within the joint 95% confidence interval and were used to calculate *R_0_*; parameter sets outside the confidence interval were discarded.

The KALANET data used to calculate the likelihood represent a situation in which the overall sand fly density is reduced on average by 12.5% (6 control clusters without reduction and 6 intervention clusters with 25% reduction; see [24]) to 87.5% by reducing the sand flies' life expectancy. To correct for this, the life expectancy values obtained in the screening process were divided by 0.875 and then set to 100% for use as non-intervention parameter values and to calculate *R_0_*.

**(b) Intervention.** To evaluate the impact of vector control measures, we simulated interventions that reduced the sand fly density either by killing sand flies (e.g., LLIN and IRS) or destroying breeding sites (e.g., EVM). To investigate how *R_e_* changed due to these interventions, the values of the corrected sand fly life expectancy, 1/*μ_F_*, and breeding site capacity, *N_F_*, were reduced systematically such that the vector density was reduced from 100 to 0%. The equilibrium sand fly density is proportional to the sand flies' life expectancy and the breeding capacity, so that we can also express the effect of interventions as a reduction in sand fly density. Because all parameter sets contained in the joint confidence intervals could be used as no-intervention starting points, we used the relative reduction of sand fly density rather than the absolute reduction to measure the effect of the interventions.

By comparing the calculated thresholds with an observed reduction in sand fly density, intervention success could be assessed in terms of *R_e_*.

## Results

**Figure 1:**
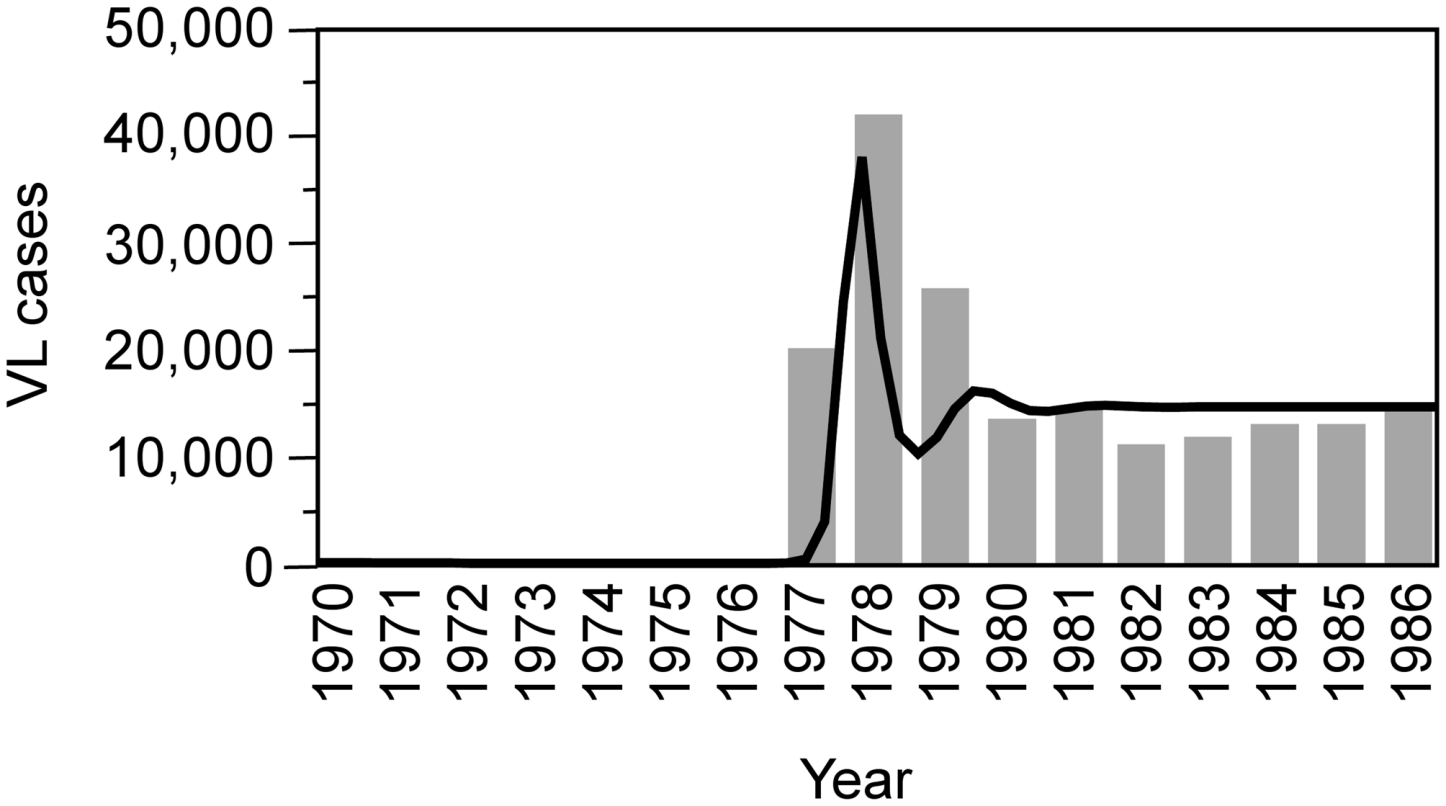
Observed and simulated re-emergence of infection dynamics after local extinction. Observed number of VL cases (grey bars) reported in India from 1970–1986 (source: Ministry of Health and Family Welfare, Government of India), together with the predicted number of cases (black curve) derived from a previously developed mathematical model [26]. As a side effect of malaria vector control, VL incidence dropped to very low levels until 1976 which has been mimicked in this simulation by reducing the sand fly population by 85% between 1967 and 1976. The residual infection predicted by the model for 1976 has been used in this simulation as seed for the observed re-introduction of VL in 1977 when the density of sand flies was assumed to return to pre-control levels because of cessation of malaria vector control measures.

Despite the fact that *L. donovani* transmission temporal or spatial heterogeneities, such as seasonality in sand fly abundance or cyclic epidemics, were not considered within this modelling approach, the model adequately reproduced the observed re-emergence of infection dynamics after DDT-based vector control, as shown in Figure 1. The observed VL incidence in Figure 1 can be reproduced by a simulation which mimics reductions in sand fly population as a consequence of indoor residual spraying against malaria between 1976 and 1977. In 1977, vector control measures were terminated abruptly and VL endemicity relapsed to pre-control levels as the sand fly population recovered. Diminished levels of immunity in the human population produce in 1978 an “overshooting” epidemic which oscillates over a period of about 4 years towards the endemic equilibrium.

**Figure 2:**
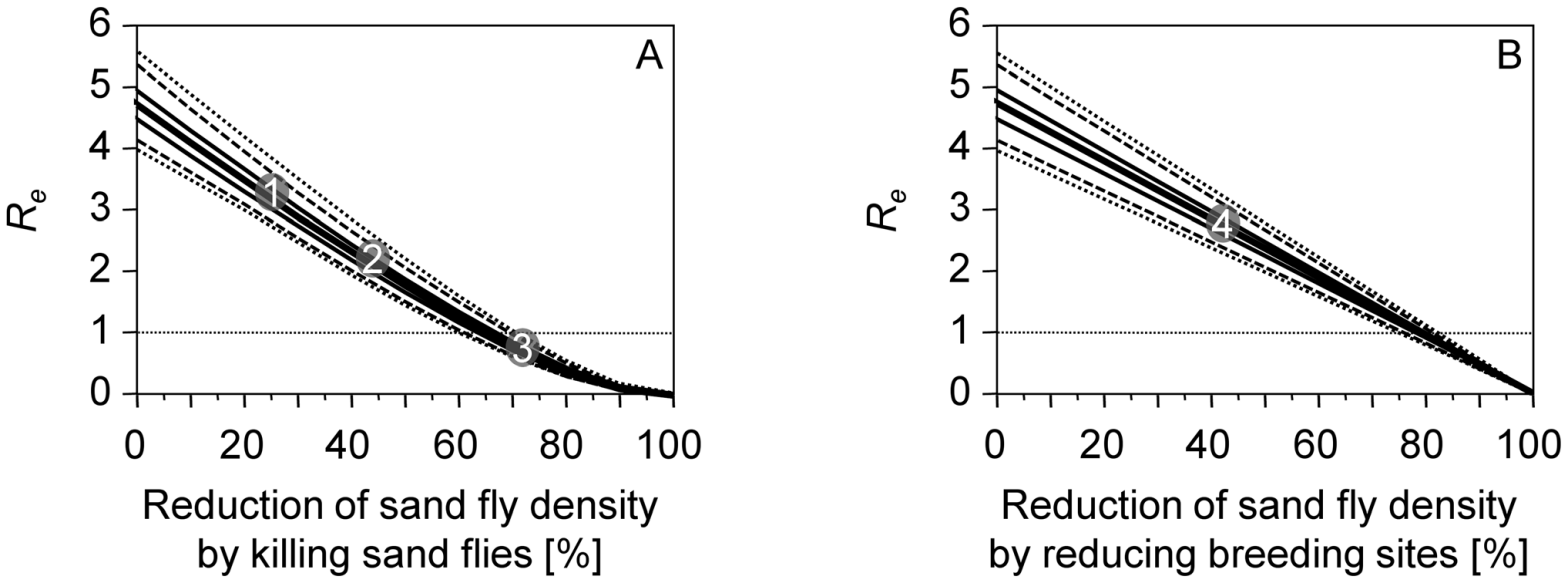
Comparison of calculated thresholds and observed reductions in sand fly density. *R_e_* estimates that depend on a reduction of sand fly density by reducing the sand flies' life expectancy (A) or their breeding site capacity (B) are represented by minimum and maximum (dotted lines), 2.5% and 97.5% quantiles (dashed lines), quartiles (thin lines) and median (bold line). Baseline sand fly density reflects the situation in the KALANET study without control measures. Observed effects of long lasting insecticidal nets (circles 1 and 2), indoor residual spraying (circle 3) and environmental management (circle 4) on the sand fly density, as reported in two recent cluster randomised controlled trials [23], [24], are displayed as grey circles.

A total of 204 out of 2,400,000 randomly generated parameter combinations fulfilled the likelihood criterion (see Methods) and were selected for the following analysis. Ranges, means and medians of the accepted parameters are provided in Table 1. The basic reproduction number of VL under the conditions observed in the KALANET project without any vector control measures was estimated as *R_0_* = 4.71 ( = median pre-study *R_0_*). Due to uncertainties in parameter estimation, considerable variation of *R_0_* is possible; without intervention, 95% of *R_0_* estimates range from 4.1 to 5.4 (Figures 2A and B at 0% sand fly reduction).

**Figure 3:**
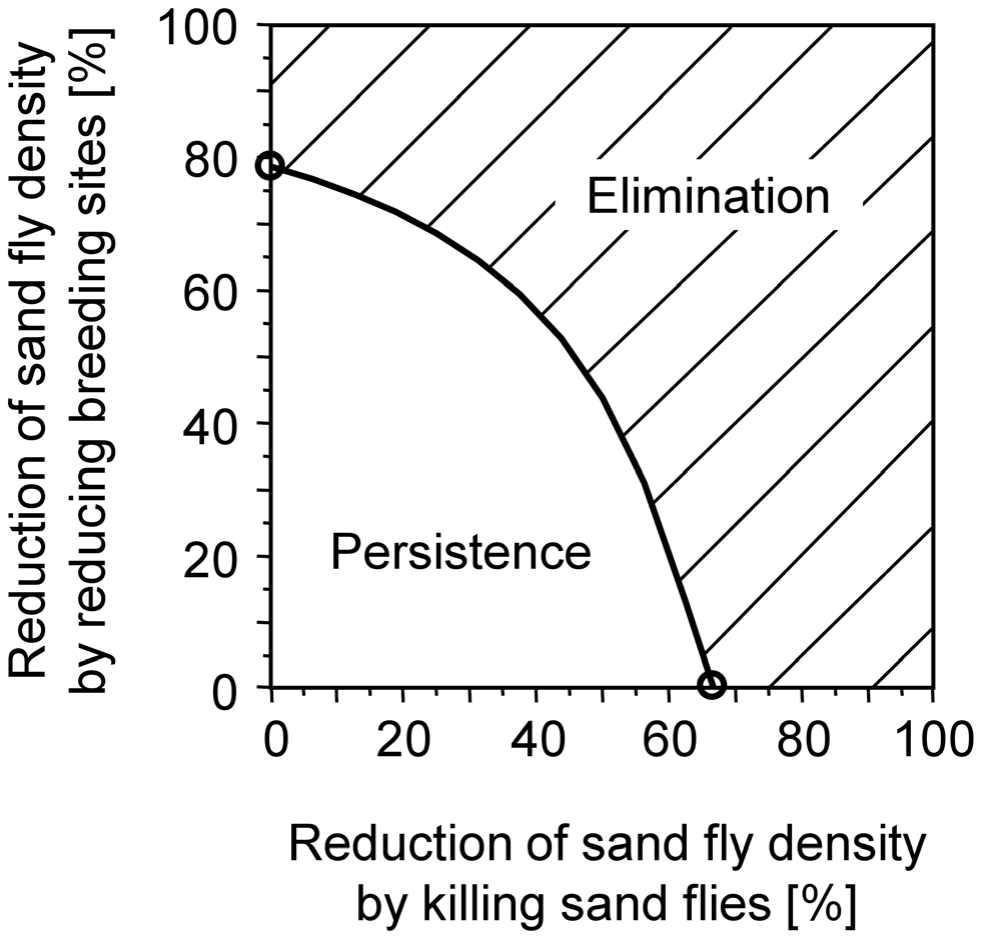
Combinations of vector control measures necessary for elimination of VL. Simulation results of combined reduction of the sand flies' life expectancy and their breeding site capacity. The dashed area above the curve represents combinations leading to *R_e_*<1 and thus allow for elimination of VL. The area below the curve represents combinations leading to *R_e_*>1 and thus imply persistence. The two circles on the axes represent the thresholds when only one intervention will be performed (cf. Figure 2: 67% reduction of fly density by reducing their life expectancy, 79% reduction in breeding site capacity). Killing of sand flies is more effective than reducing breeding site capacity because it attacks adult flies of which some are already infected.

*R_e_* is reduced non-linearly when sand fly density is reduced by killing flies and linearly when density is reduced by destroying breeding sites (Figures 2A and B). A reduction of the sand fly population large enough to interrupt endemic *L. donovani* transmission requires achieving *R_e_*<1. A reduction of sand fly density by reducing the sand flies' longevity has a different effect on *R_e_* than a reduction by means of breeding site control, even if both measures lead to the same sand fly density:

- If the sand flies' life expectancy is reduced, our analyses suggest that elimination can be achieved by reducing the sand fly density by 67%, which is a reduction of approximately two-thirds. Due to parameter uncertainties, the necessary reduction ranges from 60 to 72% (Figure 2A for *R_e_* = 1).
- If breeding sites are destroyed, elimination can be achieved by reducing the sand fly density by 79%, which is a reduction of approximately four-fifths. Due to parameter uncertainties, the necessary reduction ranges from 75 to 82% (Figure 2B for *R_e_* = 1).
- The effects of a combined intervention strategy involving both before mentioned control measures are shown in Figure 3.

Based on the calculated thresholds, we investigated the effectiveness of the three major vector-related interventions: LLIN, EVM and IRS. The effects of these interventions have recently been evaluated by two cluster randomised controlled trials [23], [24]. To assess intervention success in terms of *R_e_*, the observed reductions in the sand fly density are visualised as grey circles in Figures 2A and B. LLIN (circles 1 and 2 in Figure 2A) and EVM (circle 4 in Figure 2B) reduced *R_e_* but were not sufficient to reach *R_e_* = 1, whereas IRS (circle 3 in Figure 2A) reduced transmission to a level at which infection cannot persist. Combination of intervention strategies seems promising, the dashed area above the curve in Figure 3 represents combinations leading to *R_e_*<1 and thus allow for elimination of VL.

## Discussion

We estimated a basic reproduction number, *R_0_*, of 4.71 ( = median pre-study *R_0_*) for India and Nepal; this value is slightly higher than the previously published value of 3.94 [26], which actually referred to an effective reproduction number in a study site where the fly density was reduced to 87.5%. We determined the thresholds necessary for vector-related interventions to interrupt *L. donovani* transmission. Reduction of the vector's life expectancy is more effective than a reduction of the vector's breeding site capacity; due to the non-linear relationship between *R_e_* and life expectancy and the linear relationship between *R_e_* and breeding site capacity, a reduction of the sand fly density by 67% through killing flies leads to the same effect as a reduction by 79% through destroying breeding sites. Treated nets and to a minor extent IRS, predominantly kill sand flies that are about to transmit the infection, whereas breeding site control generally reduces the number of flies, regardless of whether they reach an age where transmission occurs.

### Long lasting insecticidal nets

The two studies investigating the effects of LLIN reported reductions in indoor sand fly density by 25% [24] and 44% [23] (Figure 2A), far below the elimination threshold of approximately 67%. These reductions correspond well with the results obtained in the KALANET trial, in which LLIN reduced the risk of *L. donovani* infection and clinical VL in India and Nepal by only 10 and 1%, respectively [21]. In the KALANET trial, compliance of study subjects with LLIN use was very good and actively monitored and encouraged [25], so inappropriate use cannot explain the lack of an observed effect.

Treated nets can be considered as baited traps that kill predominantly sand flies that are about to transmit the infection, making LLIN a highly effective intervention tool. However, three major reasons may limit the effectiveness of LLIN: (1) inappropriate usage of LLIN by man, (2) changed and/or alternative feeding or resting behaviour of the vectors and (3) vector adaptation or habituation against insecticidal substances, as described in more detail in the following paragraphs.

*P. argentipes*, the only known vector on the Indian subcontinent, is believed to live and reproduce within human dwellings and to be most active when people are asleep [30]. However, evidence of exophilic behaviour of *P. argentipes* has been shown in Bihar, where on average 30 sand flies were collected per trap per night in the palm tree canopy. Despite a height of approximately 18 m above ground, a large proportion of the flies had fed on humans [31], [32]. Therefore, the common wisdom depicting *P. argentipes* as a low-range flying or hopping insect may need to be revised. It is currently unknown whether the observed abundance of *P. argentipes* in palm tree canopies represents an adaptation of the vector on extensive indoor residual spraying or whether *P. argentipes* in general exhibits more exophilic and even exophagic behaviour than commonly assumed. If the flight range of *P. argentipes* is not as limited as previously thought, the limited effect of LLIN on *L. donovani* transmission as reported [23]–[33] may derive from an altered feeding behaviour of the vector choosing unprotected humans or animals as alternative hosts. Exophilic behaviour of malaria vectors has evolved after prolonged spraying [34].

Successful control using LLIN may depend on the regional history of spraying, the vector's behavioural or physiological adaptation or its habituation to insecticidal substances; in Bangladesh, where there was practically no vector control until 2010 [35], LLIN reduced the vector density by 70 to 80% [23], whereas LLIN was considerably less effective in India and Nepal, where vector control had been performed somewhat regularly. In a recent study in Bangladesh, VL incidence was reduced by 65% by LLIN [36]. Although the effect of LLIN on vector density was not evaluated within that study, the results corroborate the high effectiveness of LLIN in Bangladesh.

### Environmental management

Environmental management aims to impede sand flies from breeding. The focal distribution of VL implies that a number of highly specific environmental requirements must be fulfilled at the same location [6] to allow for a high local vector density. Alluvial alkaline soil and high rainfall, for example, are believed to be geographically correlated with VL foci in India [37] and *P. argentipes* is believed to breed preferably in cattle sheds built with alkaline soil [38]. Plastering houses with different materials is one measure to destroy indoor breeding sites for *P. argentipes*. In Bangladesh, plastering houses with mud did not reduce sand fly density, whereas plastering with a lime/mud mixture in India and Nepal reduced the *P. argentipes* indoor density [23]. Overall, wall plastering only reduced the sand fly density by 42%, whereas our investigations demand a reduction of 79% (Figure 2B). Nevertheless, scarce data are available on the effect of EVM. In general, destroying breeding sites of *P. argentipes* is a promising tool for intervention and should also prevent re-emergence of infection after local extinction. To improve intervention strategies aimed at breeding site control, we need better information about the living and feeding requirements of the vectors' larvae. Because we do not understand the breeding sites of *P. argentipes*, it is possible that other places, such as soil, might be involved and plastering walls may not be sufficient. The importance of breeding sites as a key target for vector control is illustrated by malaria elimination in temperate zones, which may be due largely to river regulation (e.g., the Upper Rhine by Tulla, 1817 to 1876) and other ecological interventions affecting the vector's breeding sites [39].

### Indoor residual spraying

The study conducted in India, Nepal and Bangladesh on the effects of IRS showed a reduction of the sand fly density by 72%, which exceeds the elimination threshold of 67%. The near elimination of VL in the past as a collateral benefit of the global malaria eradication programme is used as historic evidence for the high efficacy of this method. Nonetheless, IRS requires a strictly regulated approach and may suffer from poor quality assurance [40]. DDT resistance has been reported for *P. argentipes* in India since the early 1990s and was recently reported in Nepal from a village bordering Bihar [15], [41], [42]. Because of reports about high DDT levels in humans exposed to DDT indoor spraying measures and increasing evidence that DDT causes harm to humans and animals, the Stockholm Convention on persistent organic pollutants has banned DDT [43], with the exception of DDT production and use for purposes of vector control. In Nepal, DDT has been replaced by less persistent synthetic pyrethroids since 1995, in Bangladesh, vector control activities were started only very recently, but India still relies on DDT, spraying two annual rounds of DDT in VL endemic districts [42]. The benefit of IRS with DDT or pyrethroids still seems to outweigh the harms of this measure [43]. However, resistance against DDT continues to spread and cross-resistance may emerge (e.g., in anophelines, the so-called knockdown resistance, a DDT/pyrethroid cross-resistance, is commonly found [44]). Thus, IRS may only be a transient measure to effectively reduce sand fly density.

### Integrated vector management

Integrated vector management, which combines different vector control measures, could be an effective approach to overcome the limitations of the different tools applied independently. For instance, a reduction in the breeding site capacity by 42% by wall plastering would require an additional reduction of the sand fly density by 50% by IRS to exceed the elimination threshold (see Figure 3). If more is known about *P. argentipes*' breeding behaviour, suitable environmental management may complement IRS. More targeted insecticide applications that take advantage of sugar meals on plants or blood meals on domestic animals or that act specifically against sand fly larvae may increase insecticide efficacy. Such interventions have been developed at the prototype stage [45], [46] but should be further evaluated. Whether combinations of vector control measures will have a stronger effect on the sand fly population or even reinforce each other requires further investigation.

### Uncertainties and restrictions

In our analysis, uncertainty in parameter estimation translates into uncertainty of *R_e_*. Additional variability can emerge when further parameters such as temporal or spatial heterogeneities, which were not considered in our approach, are included. Additional restrictions are that (i) our model was calibrated to average prevalences under the assumption of homogeneous spread of the infection among humans and vectors, (ii) animals serving as alternative blood hosts were not considered and (iii) the infection probabilities for susceptible flies when feeding on a VL- or PKDL-patient and for susceptible humans after being the blood meal of an infected sand fly are assumed to be 100%. Data on infection rates in sand flies and humans are scarce. One study showed infection rates between 0.5 and 5% for *P. argentipes* fed on VL-patients [47]. For *P. perniciosus* fed on HIV co-infected VL-patients, infection rates range from 9 to 89% [48] and for *P. perniciosus* fed on dogs, infection rates range from 40 to 100% [49]. To validate model predictions and to reduce uncertainties, more data on infection rates are urgently needed. In addition, studies simultaneously investigating *L. donovani* infection in the human and sand fly population are required. Animals serving as alternative blood hosts or even reservoirs [50] should be investigated and the contribution of asymptomatically infected humans in *L. donovani* transmission requires more attention.

The deterministic modelling approach should be extended to an individual-based, stochastic model which can consider, for instance, the factors heterogeneity in exposure, spatial stratifications and temporal dynamics in VL incidence which are important limitations of the current modelling approach.

### Conclusions

Previous modelling analyses suggest that transmission of *L. donovani* is predominantly driven by asymptomatically infected hosts. Thus, in contrast to treatment, vector control has the potential to eliminate VL [26]. However, to prevent re-emergence of infection after local extinction in formerly endemic regions, low vector densities should be maintained and combined with active case detection in humans as well as effective treatment. Only a few parasites, hidden in humans after being putatively cured (cases of PKDL may appear years after infection), are needed to initiate the next epidemic.

In this paper, we show the limitations of currently available vector control tools. By using a previously published VL transmission model, the basic reproduction number, *R_0_*, of VL in India and Nepal is estimated as *R_0_* = 4.71 and the thresholds below which the *L. donovani* infection in the human population cannot persist are identified: a reduction of sand fly density by 79% through destroying breeding sites leads to the same effect as a reduction by 67% through killing the flies.

Elimination should be feasible because the recently evaluated effect of IRS exceeds the threshold (sand fly density reduced by approximately 72% [23]). Although the benefits of IRS currently outweigh the risks of this measure to humans and animals, emerging insecticide resistance may change this benefit/risk ratio. The observed effects of LLIN (sand fly density reduced by approximately 44% or 25% [23], [24]) and EVM (sand fly density reduced by approximately 42% [23]) do not seem to be sufficient to reach either threshold. A better understanding of the living and feeding requirements of the vectors' larvae might improve EVM.

Integrated vector management based on IRS combined with LLIN and more effective EVM may allow overcoming the limitations of the current vector control methods and should also prevent re-emergence of the infection after local extinction. To keep sand fly density low, new tools targeting the immature stages of sand flies or the exophilic and zoophagic behaviour of *P. argentipes* should be developed.

## Supporting Information

Figure S1 **Model for Leishmania donovani infection, transmission and control.** Compartments represent proportions of humans and vectors distinguished (horizontally) according to their history of infection (defined by diagnostic states). The diagnostics comprised PCR, DAT and LST with combinations shown in the bar on the top of the graph. Human hosts are further distinguished (vertically) by disease and treatment status. For further variables and parameters, see Text S1 and Tables S1 to S6 in the Supplement.
(TIF)

Table S1 **Model variables – sand flies.**
(DOC)

Table S2 **Model variables – humans.**
(DOC)

Table S3 **Model parameters – sand flies.** ([51]–[53]).
(DOC)

Table S4 **Model parameters – humans.** ([54]–[56]).
(DOC)

Table S5 **Model parameters – treatment.** ([57]–[61]).
(DOC)

Table S6 **Model parameters – immuno-compromised humans.** ([62]).
(DOC)

Text S1 **Supplemental text.** Model equations are provided together with an expression for the effective reproduction number *R_e_*.
(DOC)
