# Supplementary figures and images for: Model-Based Investigations of Different Vector-Related Intervention Strategies to Eliminate Visceral Leishmaniasis on the Indian Subcontinent
Source: PLoS Negl Trop Dis. 2014 Apr 24;8(4):e2810. doi: 10.1371/journal.pntd.0002810 (PMC3998939; doi:10.1371/journal.pntd.0002810)

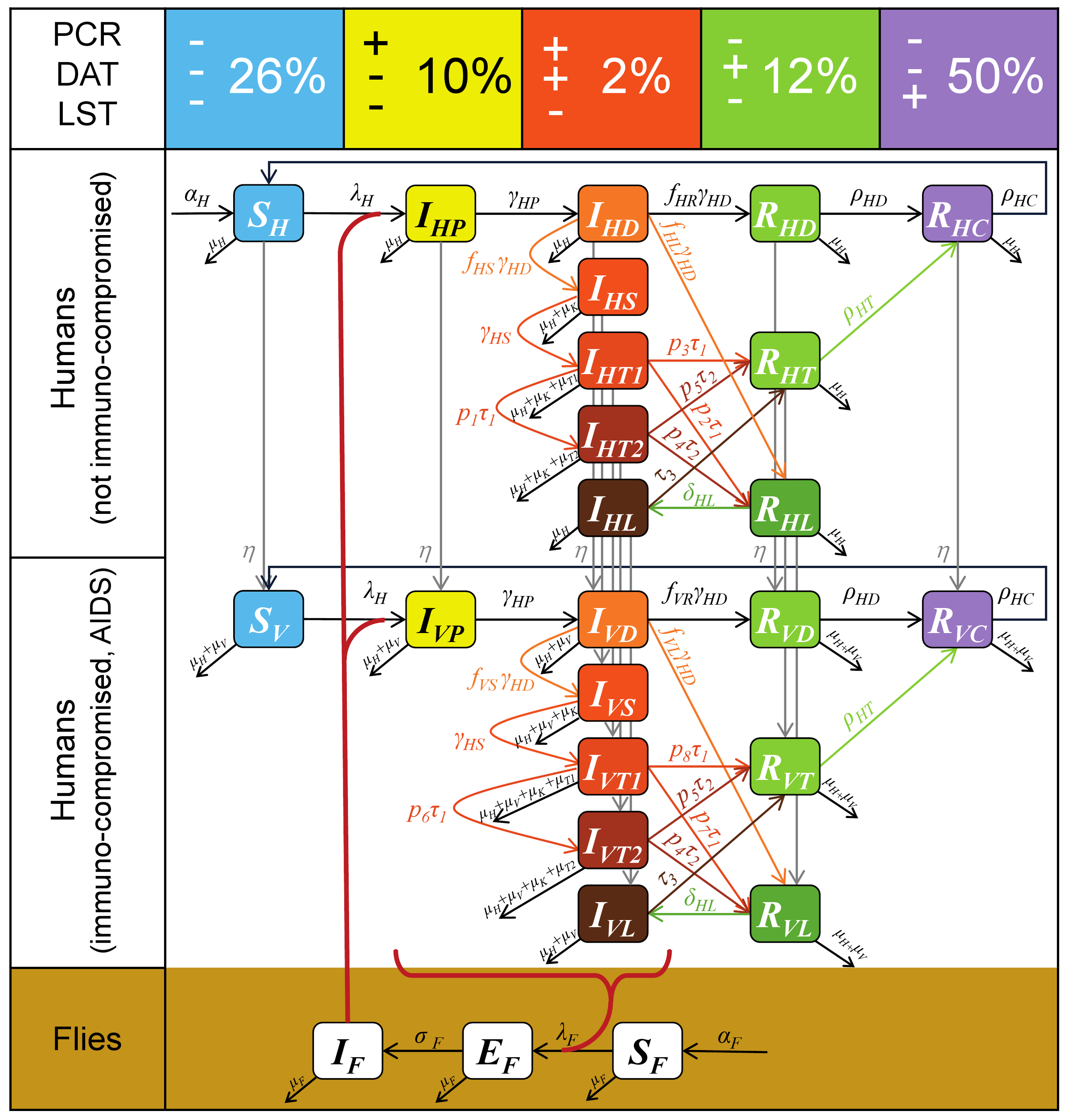

Supplement: Figure S1 — Model for Leishmania donovani infection, transmission and control. Compartments represent proportions of humans and vectors distinguished (horizontally) according to their history of infection (defined by diagnostic states). The diagnostics comprised PCR, DAT and LST with combinations shown in the bar on the top of the graph. Human hosts are further distinguished (vertically) by disease and treatment status. For further variables and parameters, see Text S1 and Tables S1 to S6 in the Supplement. (TIF) [file pntd.0002810.s001.tif]
