# Supplementary material for: Model-Based Investigations of Different Vector-Related Intervention Strategies to Eliminate Visceral Leishmaniasis on the Indian Subcontinent
Source: PLoS Negl Trop Dis. 2014 Apr 24;8(4):e2810. doi: 10.1371/journal.pntd.0002810 (PMC3998939; doi:10.1371/journal.pntd.0002810)
Supplement: Table S1 — Model variables – sand flies. (DOC) [file pntd.0002810.s002.doc]

Table S1 – Model variables – sand flies.

| *S_F_* | Number of sand flies in the susceptible stage |
| --- | --- |
| *E_F_* | Number of sand flies in the latent stage |
| *I_F_* | Number of sand flies in the infectious stage |
