# Supplementary material for: Model-Based Investigations of Different Vector-Related Intervention Strategies to Eliminate Visceral Leishmaniasis on the Indian Subcontinent
Source: PLoS Negl Trop Dis. 2014 Apr 24;8(4):e2810. doi: 10.1371/journal.pntd.0002810 (PMC3998939; doi:10.1371/journal.pntd.0002810)
Supplement: Table S2 — Model variables – humans. (DOC) [file pntd.0002810.s003.doc]

Table S2 – Model variables – humans.

| *S_H_*, *S_V_* | Number of humans in the susceptible stage |
| --- | --- |
| *I_HP_*, *I_VP_* | Number of humans in the early asymptomatic infectious stage |
| *I_HD_*, *I_VD_* | Number of humans in the late asymptomatic infectious stage |
| *R_HD_*, *R_VD_* | Number of humans in the early recovery stage |
| *R_HC_*, *R_VC_* | Number of humans in the late recovery stage |
| *I_HS_*, *I_VS_* | Number of humans with symptomatic VL eligible for treatment |
| *I_HT1_*, *I_VT1_* | Number of humans under first-line VL treatment |
| *I_HT2_*, *I_VT2_* | Number of humans under second-line VL treatment |
| *R_HT_*, *R_VT_* | Number of humans that recovered after treatment |
| *R_HL_*, *R_VL_*, | Number of humans putatively recovered after VL treatment but will develop PKDL |
| *I_HL_*, *I_VL_* | Number of humans with PKDL |

For immuno-compromised humans, index *H* is replaced by index *V*.
