# Supplementary material for: Model-Based Investigations of Different Vector-Related Intervention Strategies to Eliminate Visceral Leishmaniasis on the Indian Subcontinent
Source: PLoS Negl Trop Dis. 2014 Apr 24;8(4):e2810. doi: 10.1371/journal.pntd.0002810 (PMC3998939; doi:10.1371/journal.pntd.0002810)
Supplement: Table S3 — Model parameters – sand flies. ([51]–[53]). (DOC) [file pntd.0002810.s004.doc]

Table S3 – Model parameters – sand flies.

|  | Description | Reference |
| --- | --- | --- |
| *N_F_* | Breeding site capacity^§^: *N_F_* = 7,344 | Estimated |
| *µ_F_* | Mortality rate of sand flies, derived from life expectancy of sand flies: 1/µ*_F_* = 14 days |  |
| *α_F_* | Birth rate of sand flies: *α_F_* = *µ_F_* *N_F_* | Assumed |
| *σ_F_* | Rate determining the sojourn time of sand flies in stage *E_F_*, derived from 1/(*σ_F_* + *µ_F_*) = 5 days |  |
| *β* | Rate determining the feeding cycle duration, derived from 1/(*β* + *µ_F_*) = 4 days |  |

^§^ Previously referred to as ‘number of vectors’, which is correct as long as the sand flies’ population size remains constant.
