# Supplementary material for: Model-Based Investigations of Different Vector-Related Intervention Strategies to Eliminate Visceral Leishmaniasis on the Indian Subcontinent
Source: PLoS Negl Trop Dis. 2014 Apr 24;8(4):e2810. doi: 10.1371/journal.pntd.0002810 (PMC3998939; doi:10.1371/journal.pntd.0002810)
Supplement: Table S4 — Model parameters – humans. ([54]–[56]). (DOC) [file pntd.0002810.s005.doc]

Table S4 – Model parameters – humans.

|  | Description | Reference |
| --- | --- | --- |
| *N_H_* | Number of humans: *N_H_* = 1,000 | Population size |
| *µ_H_* | Mortality rate of humans, derived from baseline life expectancy of humans: 1/*µ_H_* = 40 years | Assumed |
| *α_H_* | Birth rate of humans, chosen such that the human population size remains constant throughout the simulation (i.e., the number of births equals the number of deaths at any time) | Assumed |
| *µ_K_* | Excess mortality rate caused by VL assumed to be *µ_K_* = 0.2/month | Assumed |
| *p_H_* | Probability that a human becomes infected after being the blood meal of an infected sand fly: *p_H_* = 1 | Assumed |
| *p_F2_* | Probability that a susceptible fly becomes infected when feeding on a human host in stage *I_HD_* or *I_VD_*: *p_F2_* = 0.0417 | Estimated |
| *p_F1_* | Probability that a susceptible fly becomes infected when feeding on a human host in stage *I_HP_* or *I_VP_*: *p_F1_* = *p_F2_*/2 | Assumed |
| *p_F3_* | Probability that a susceptible fly becomes infected when feeding on a VL patient in stage *I_HS_*, *I_HT1_*, *I_HT2_*, *I_VS_*, *I_VT1_* or *I_VT2_*: *p_F3_* = 1 | Assumed |
| *p_F4_* | Probability that a susceptible fly becomes infected when feeding on a PKDL patient in stage *I_HL_* or *I_VL_*: *p_F4_* = 1 | Assumed |
| *f_HS_* | Fraction of individuals in stage *I_HD_* who develop symptomatic VL: *f_HS_* = 0.0028 | Estimated |
| *f_HL_* | Fraction of individuals in stage *I_HD_* who go directly to *R_HL_* and will later develop PKDL: *f_HL_* = 0.0001 |  |
| *f_HR_* | Fraction of individuals in stage *I_HD_* who recover without showing a symptomatic course of infection (→*R_HD_*): *f_HR_* = 1–(*f_HS_*+*f_HL_*) |  |
| *γ_HP_* | Rate determining the sojourn time in the early asymptomatic stage *I_HP_*, derived from 1/(*γ_HP_*+*µ_H_*) = 60 days |  |
| *γ_HD_* | Rate determining the sojourn time in the late asymptomatic stage *I_HD_*, derived from 1/(*γ_HD_*+*µ_H_*) = 12 days | Estimated |
| *γ_HS_* | Rate determining the duration between diagnosis of VL and onset of treatment, derived from 1/(*γ_HS_*+*µ_H_*+*µ_K_*) = 1 day | Conditions in the KALANET trial |
| *ρ_HD_* | Rate determining the period of DAT-positivity in stage *R_HD_*, derived from 1/(*ρ_HD_*+*µ_H_*) = 74 days | Estimated |
| *ρ_HT_* | Rate determining the period of DAT-positivity in stage *R_HT_*, derived from 1/(*ρ_HT_*+*µ_H_*) = 1/(*ρ_HD_*+*µ_H_*) | Assumed |
| *ρ_HC_* | Rate determining the period of LST-positivity in stage *R_HC_*, derived from 1/(*ρ_HC_*+*µ_H_*) = 308 days | Estimated |
