# Supplementary material for: Model-Based Investigations of Different Vector-Related Intervention Strategies to Eliminate Visceral Leishmaniasis on the Indian Subcontinent
Source: PLoS Negl Trop Dis. 2014 Apr 24;8(4):e2810. doi: 10.1371/journal.pntd.0002810 (PMC3998939; doi:10.1371/journal.pntd.0002810)
Supplement: Table S5 — Model parameters – treatment. ([57]–[61]). (DOC) [file pntd.0002810.s006.doc]

Table S5 – Model parameters – treatment.

|  | Description | Reference |
| --- | --- | --- |
| *τ_1_* | Rate determining the sojourn time under first-line VL treatment, derived from 1/(*τ_1_*+*µ_H_*+*µ_K_*) = 30 days |  |
| *τ_2_* | Rate determining the sojourn time under second-line VL treatment, derived from 1/(*τ_2_*+*µ_H_*+*µ_K_*) = 30 days |  |
| *τ_3_* | Rate determining the sojourn time under PKDL treatment, derived from 1/(*τ_3_*+*µ_H_*) = 180 days |  |
| *µ_T1_* | Excess mortality rate caused by first-line VL treatment, derived from *µ_T1_* = *f_T_* *τ_1_*, assuming that *f_T_* = 5% die because of treatment |  |
| *µ_T2_* | Excess mortality rate caused by second-line VL treatment, derived from *µ_T2_* = *f_T_* *τ_2_*, assuming that *f_T_* = 5% die because of treatment |  |
| *p_1_* | Proportion of VL patients not responding to VL first-line treatment, derived from *p_1_* = (1-*f_T_*) *f_1_*, assuming that *f_1_* = 5% of VL patients who are not killed by the treatment and do not respond to VL first-line treatment |  |
| *p_2_* | Proportion of VL patients who appear to recover under VL first-line treatment but will later develop PKDL, derived from  *p_2_* = (1-*f_T_*)(1-*f_1_*) *f_2_*, assuming that a fraction *f_2_* = 3% of VL patients who are neither killed by the treatment nor experienced obvious treatment failure and appeared to recover under VL treatment but will develop PKDL |  |
| *p_3_* | Proportion of VL patients recovering during VL first-line treatment, derived from *p_3_* = (1-*f_T_*)(1-*f_1_*)(1-*f_2_*) |  |
| *p_4_* | Proportion of VL patients who appear to recover under VL second-line treatment but will later develop PKDL, derived from  *p_4_* = (1-*f_T_*) *f_2_*, assuming that a fraction *f_2_* = 3% of VL patients who are not killed by the treatment and appeared to recover under VL treatment but will develop PKDL |  |
| *p_5_* | Proportion of VL patients recovering during VL second-line treatment, derived from *p_5_* = (1-*f_T_*)(1-*f_2_*) |  |
| *δ_HL_* | Rate determining the sojourn time in stage *R_HL_*, derived from 1/(*δ_HL_*+*µ_H_*) = 21 months |  |
