# Supplementary material for: Model-Based Investigations of Different Vector-Related Intervention Strategies to Eliminate Visceral Leishmaniasis on the Indian Subcontinent
Source: PLoS Negl Trop Dis. 2014 Apr 24;8(4):e2810. doi: 10.1371/journal.pntd.0002810 (PMC3998939; doi:10.1371/journal.pntd.0002810)
Supplement: Text S1 — Supplemental text. Model equations are provided together with an expression for the effective reproduction number Re. (DOC) [file pntd.0002810.s008.doc]

# Supplemental text

## Equations

### Humans

$$\begin{matrix} \frac{dS_{H}}{dt}=\alpha_{H}+\rho_{HC}R_{HC}-\left(\lambda_{H}+\mu_{H}+\eta\right)S_{H} \\ \frac{dI_{HP}}{dt}=\lambda_{H}S_{H}-\left(\gamma_{HP}+\mu_{H}+\eta\right)I_{HP} \\ \frac{dI_{HD}}{dt}=\gamma_{HP}I_{HP}-\left(\gamma_{HD}+\mu_{H}+\eta\right)I_{HD} \\ \frac{dR_{HD}}{dt}=f_{HR}\gamma_{HD}I_{HD}-\left(\rho_{HD}+\mu_{H}+\eta\right)R_{HD} \\ \frac{dR_{HC}}{dt}=\rho_{HD}R_{HD}+\rho_{HT}R_{HT}-\left(\rho_{HC}+\mu_{H}+\eta\right)R_{HC} \\ \frac{dI_{HS}}{dt}=f_{HS}\gamma_{HD}I_{HD}-\left(\gamma_{HS}+\mu_{H}+\mu_{K}+\eta\right)I_{HS} \\ \frac{dI_{HT1}}{dt}=\gamma_{HS}I_{HS}-\left(\left(1-f_{T}\right)\tau_{1}+\mu_{H}+\mu_{K}+\mu_{T1}+\eta\right)I_{HT1} \\ \frac{dR_{HT}}{dt}=p_{3}\tau_{1}I_{HT1}+p_{5}\tau_{2}I_{HT2}+\tau_{3}I_{HL}-\left(\rho_{HT}+\mu_{H}+\eta\right)R_{HT} \\ \frac{dI_{HT2}}{dt}=p_{1}\tau_{1}I_{HT1}-\left(\left(1-f_{T}\right)\tau_{2}+\mu_{H}+\mu_{K}+\mu_{T2}+\eta\right)I_{HT2} \\ \frac{dR_{HL}}{dt}=p_{2}\tau_{1}I_{HT1}+p_{4}\tau_{2}I_{HT2}+f_{HL}\gamma_{HD}I_{HD}-\left(\delta_{HL}+\mu_{H}+\eta\right) \\ \frac{dI_{HL}}{dt}=\delta_{HL}R_{HL}-\left(\tau_{3}+\mu_{H}+\eta\right)I_{HL} \end{matrix}$$

whereby $\mu_{T1}=f_{T}\cdot\tau_{1}$ and $\mu_{T2}=f_{T}\cdot\tau_{2}$

### Immuno-compromised humans

$\begin{matrix} \frac{dS_{V}}{dt}=\eta S_{H}+\rho_{HC}R_{VC}-\left(\lambda_{H}+\mu_{H}+\mu_{V}\right)S_{V} \\ \frac{dI_{VP}}{dt}=\eta I_{HP}+\lambda_{H}S_{V}-\left(\gamma_{HP}+\mu_{H}+\mu_{V}\right)I_{VP} \\ \frac{dI_{VD}}{dt}=\eta I_{HD}+\gamma_{HP}I_{VP}-\left(\gamma_{HD}+\mu_{H}+\mu_{V}\right)I_{VD} \\ \frac{dR_{VD}}{dt}=\eta R_{HD}+f_{VR}\gamma_{HD}I_{VD}-\left(\rho_{HD}+\mu_{H}+\mu_{V}\right)R_{VD} \\ \frac{dR_{VC}}{dt}=\eta R_{HC}+\rho_{HD}R_{VD}+\rho_{HT}R_{VT}-\left(\rho_{HC}+\mu_{H}+\mu_{V}\right)R_{VC} \\ \frac{dI_{VS}}{dt}=\eta I_{HS}+f_{VS}\gamma_{HD}I_{VD}-\left(\gamma_{HS}+\mu_{H}+\mu_{K}+\mu_{V}\right)I_{VS} \\ \frac{dI_{VT1}}{dt}=\eta I_{HT1}+\gamma_{HS}I_{VS}-\left(\left(1-f_{T}\right)\tau_{1}+\mu_{H}+\mu_{K}+\mu_{T1}+\mu_{V}\right)I_{VT1} \\ \frac{dR_{VT}}{dt}=\eta R_{HT}+p_{7}\tau_{1}I_{VT1}+p_{5}\tau_{2}I_{VT2}+\tau_{3}I_{VL}-\left(\rho_{HT}+\mu_{H}+\mu_{V}\right)R_{VT} \\ \frac{dI_{VT2}}{dt}=\eta I_{HT2}+p_{6}\tau_{1}I_{VT1}-\left(\left(1-f_{T}\right)\tau_{2}+\mu_{H}+\mu_{K}+\mu_{T2}+\mu_{V}\right)I_{VT2} \\ \frac{dR_{VL}}{dt}=\eta R_{HL}+p_{2}\tau_{1}I_{VT1}+p_{4}\tau_{2}I_{VT2}+f_{VL}\gamma_{HD}I_{VD}-\left(\delta_{HL}+\mu_{H}+\mu_{V}\right)R_{VL} \\ \frac{dI_{VLs}}{dt}=\eta I_{HLs}+\delta_{HL}R_{VLs}-\left(\tau_{3}+\mu_{H}+\mu_{V}\right)I_{VLs} \end{matrix}$

whereby $\mu_{T1}=f_{T}\cdot\tau_{1}$ and $\mu_{T2}=f_{T}\cdot\tau_{2}$ and

$$\begin{matrix} \alpha_{H}=\mu_{H}N_{H} \\ +\mu_{K}\left(I_{HS}+I_{HT1}+I_{HT2}+I_{VS}+I_{VT1}+I_{VT2}\right) \\ +\mu_{V}\left(S_{V}+I_{VP}+I_{VD}+R_{VD}+R_{VC}+I_{VS}+I_{VT1}+I_{VT2}+R_{VT}+R_{VL}+I_{VL}\right) \\ +\mu_{T1}\left(I_{HT1}+I_{VT1}\right) \\ +\mu_{T2}\left(I_{HT2}+I_{VT2}\right) \end{matrix}$$

### Sand flies

$$\begin{matrix} \frac{dS_{F}}{dt}=\alpha_{F}-\left(\lambda_{F}+\mu_{F}\right)S_{F} \\ \frac{dE_{F}}{dt}=\lambda_{F}S_{F}-\left(\sigma_{F}+\mu_{F}\right)E_{F} \\ \frac{dI_{F}}{dt}=\sigma_{F}E_{F}-\mu_{F}I_{F} \end{matrix}$$

### Infection rate λ

$$\begin{matrix} \lambda_{H}=\frac{\beta\cdot p_{H}\cdot I_{F}}{N_{H}} \\ \lambda_{F}=\frac{\beta\cdot p_{F1}\left(I_{HP}+I_{VP}\right)+p_{F2}\left(I_{HD}+I_{VD}\right)+p_{F3}\left(I_{HS}+I_{HT1}+I_{HT2}\right)+p_{F4}I_{HL}+p_{F5}\left(I_{VS}+I_{VT1}+I_{VT2}\right)+p_{F6}I_{VL}}{N_{H}} \end{matrix}$$

## Calculation of the basic reproduction number R_0_

*R_0_* is calculated from the next generation matrix that considers the four combinations of infections between humans who are HIV-positive or -negative with several elements, as follows: *a*: infections of HIV-negative humans via flies infected by HIV-negative humans, *b*: HIV-positive humans infected via flies infected by HIV-negative humans, *c*: HIV-negative humans infected via flies infected by HIV-positive humans, *d*: infections of HIV-positive humans via flies infected by HIV-positive humans. Using these elements, the next generation matrix is given by

$M=\left(\begin{matrix} a & b \\ c & d \end{matrix}\right)$.

For purposes of comprehensive mathematical notation we use the following abbreviations:

*D*'s denote mean sojourn times in infectious compartments as denoted by the corresponding indices and *f*'s denote the fractions of infected humans who undergo transition to the downstream compartment.

For humans not infected with HIV, these are:

$D_{HP}=\frac{1}{\gamma_{HP}+\mu_{H}+\eta}$, $D_{HD}=\frac{1}{\gamma_{HD}+\mu_{H}+\eta}$, $D_{HS}=\frac{1}{\gamma_{HS}+\mu_{H}+\mu_{K}+\eta}$, $D_{HT1}=\frac{1}{\tau_{1}+\mu_{H}+\mu_{K}+\eta}$, $D_{HT2}=\frac{1}{\tau_{2}+\mu_{H}+\mu_{K}+\eta}$, $D_{RHL}=\frac{1}{\delta_{HL}+\mu_{H}+\eta}$ and $D_{HL}=\frac{1}{\tau_{3}+\mu_{H}+\eta}$.

$f_{HP}=\gamma_{HP}\cdot D_{HP}$, $f_{HD}=f_{HS}\cdot\gamma_{HD}\cdot D_{HD}$, $f_{HD2}=f_{HL}\cdot\gamma_{HD}\cdot D_{HD}$, $f_{H\_S}=\gamma_{HS}\cdot D_{HS}$, $f_{HT1}=p_{1}\cdot\tau_{1}\cdot D_{HT1}$, $f_{HT12}=p_{2}\cdot\tau_{1}\cdot D_{HT1}$, $f_{HT2}=p_{4}\cdot\tau_{2}\cdot D_{HT2}$ and $f_{RHL}=\delta_{HL}\cdot D_{RHL}$.

For humans infected with HIV, these are:

$D_{VP}=\frac{1}{\gamma_{HP}+\mu_{H}+\mu_{V}}$, $D_{VD}=\frac{1}{\gamma_{HD}+\mu_{H}+\mu_{V}}$, $D_{VS}=\frac{1}{\gamma_{HS}+\mu_{H}+\mu_{K}+\mu_{V}}$, $D_{VT1}=\frac{1}{\tau_{1}+\mu_{H}+\mu_{K}+\mu_{V}}$, $D_{VT2}=\frac{1}{\tau_{2}+\mu_{H}+\mu_{K}+\mu_{V}}$, $D_{RVL}=\frac{1}{\delta_{HL}+\mu_{H}+\mu_{V}}$ and $D_{VL}=\frac{1}{\tau_{3}+\mu_{H}+\mu_{V}}$.

$f_{VP}=\gamma_{HP}\cdot D_{VP}$, $f_{VD}=f_{VS}\cdot\gamma_{HD}\cdot D_{VD}$, $f_{VD2}=f_{VL}\cdot\gamma_{HD}\cdot D_{VD}$, $f_{VS}=\gamma_{HS}\cdot D_{VS}$, $f_{VT1}=p_{6}\cdot\tau_{1}\cdot D_{VT1}$, $f_{VT12}=p_{7}\cdot\tau_{1}\cdot D_{VT1}$, $f_{VT2}=p_{4}\cdot\tau_{2}\cdot D_{VT2}$ and $f_{RVL}=\delta_{HL}\cdot D_{RVL}$.

In the following equations, *s* denotes the sand fly part that is similar for all compartments and is given by $s=\left(\frac{\beta}{N_{H}}\right)^{2}\cdot p_{H}\cdot N_{F}\cdot\frac{\sigma_{F}}{\sigma_{F}+\mu_{F}}\cdot\frac{1}{\mu_{F}}\cdot N_{H}$. Furthermore, the prevalence of HIV in the study area is *P_V_*= 0.003 (see Table S6).

With these notations, element *a* in matrix *M* is given by

$$a=R_{IHP\_1}+R_{IHD\_1}+R_{IHS\_1}+R_{IHT1\_1}+R_{IHT2\_1}+R_{IHL\_1}$$

whereby

$$\begin{matrix} R_{IHP\_1}=\left(D_{HP}\cdot p_{F1}+\eta\cdot D_{HP}\left(D_{VP}\cdot p_{F1}+f_{VP}\left(D_{VD}\cdot p_{F2}+f_{VD}\left(\begin{matrix} D_{VS}\cdot p_{F3}+f_{VS} \\ \left(\begin{matrix} D_{VT1}\cdot p_{F3}+f_{VT1} \\ \left(\begin{matrix} D_{VT2}\cdot p_{F3} \\ +f_{VT2}\cdot f_{RVL}\cdot D_{VL}\cdot p_{F4} \end{matrix}\right) \end{matrix}\right) \end{matrix}\right)\right)\right)\right)s\left(1-P_{V}\right) \\ +\eta\cdot D_{HP}\cdot f_{VP}\cdot f_{VD2}\cdot f_{RVL}\cdot D_{VL}\cdot p_{F4}\cdot s\left(1-P_{V}\right) \\ +\eta\cdot D_{HP}\cdot f_{VP}\cdot f_{VD}\cdot f_{VS}\cdot f_{VT12}\cdot f_{RVL}\cdot D_{VL}\cdot p_{F4}\cdot s\left(1-P_{V}\right) \end{matrix}$$

$$\begin{matrix} R_{IHD\_1}=f_{HP}\left(D_{HD}\cdot p_{F2}+\eta\cdot D_{HD}\left(D_{VD}\cdot p_{F2}+f_{VD}\left(D_{VS}\cdot p_{F3}+f_{VS}\left(\begin{matrix} D_{VT1}\cdot p_{F3}+f_{VT1} \\ \left(\begin{matrix} D_{VT2}\cdot p_{F3} \\ +f_{VT2}\cdot f_{RVL}\cdot D_{VL}\cdot p_{F4} \end{matrix}\right) \end{matrix}\right)\right)\right)\right)s\left(1-P_{V}\right) \\ +\eta\cdot D_{HD}\cdot f_{VD2}\cdot f_{RVL}\cdot D_{VL}\cdot p_{F4}\cdot s\left(1-P_{V}\right) \\ +\eta\cdot D_{HD}\cdot f_{VD}\cdot f_{VS}\cdot f_{VT12}\cdot f_{RVL}\cdot D_{VL}\cdot p_{F4}\cdot s\left(1-P_{V}\right) \end{matrix}$$

$$\begin{matrix} R_{IHS\_1}=f_{HP}\cdot f_{HD}\left(D_{HS}\cdot p_{F3}+\eta\cdot D_{HS}\left(D_{VS}\cdot p_{F3}+f_{VS}\left(D_{VT1}\cdot p_{F3}+f_{VT1}\left(\begin{matrix} D_{VT2}\cdot p_{F3} \\ +f_{VT2}\cdot f_{RVL}\cdot D_{VL}\cdot p_{F4} \end{matrix}\right)\right)\right)\right)s\left(1-P_{V}\right) \\ +\eta\cdot D_{HS}\cdot f_{VS}\cdot f_{VT12}\cdot f_{RVL}\cdot D_{VL}\cdot p_{F4}\cdot s\left(1-P_{V}\right) \end{matrix}$$

$$\begin{matrix} R_{IHT1\_1}=f_{HP}\cdot f_{HD}\cdot f_{H\_S}\left(D_{HT1}\cdot p_{F3}+\eta\cdot D_{HT1}\left(D_{VT1}\cdot p_{F3}+f_{VT1}\left(D_{VT2}\cdot p_{F3}+f_{VT2}\cdot f_{RVL}\cdot D_{VL}\cdot p_{F4}\right)\right)\right)s\left(1-P_{V}\right) \\ +\eta\cdot D_{HT1}\cdot f_{VT12}\cdot f_{RVL}\cdot D_{VL}\cdot p_{F4}\cdot s\left(1-P_{V}\right) \end{matrix}$$

$$R_{IHT2\_1}=f_{HP}\cdot f_{HD}\cdot f_{H\_S}\cdot f_{HT1}\left(D_{HT2}\cdot p_{F3}+\eta\cdot D_{HT2}\left(D_{VT2}\cdot p_{F3}+f_{VT2}\cdot f_{RVL}\cdot D_{VL}\cdot p_{F4}\right)\right)s\left(1-P_{V}\right)$$

$$\begin{matrix} R_{IHL\_1}=f_{HP}\cdot f_{HD}\cdot f_{H\_S}\cdot f_{HT1}\cdot f_{HT2}\left(f_{RHL}\left(D_{HL}+\eta\cdot D_{HL}\cdot D_{VL}\right)+\eta\cdot D_{RHL}\cdot f_{RVL}\cdot D_{VL}\right)p_{F4}\cdot s\left(1-P_{V}\right) \\ +f_{HP}\cdot f_{HD2}\left(f_{RHL}\left(D_{HL}+\eta\cdot D_{HL}\cdot D_{VL}\right)+\eta\cdot D_{RHL}\cdot f_{RVL}\cdot D_{VL}\right)p_{F4}\cdot s\left(1-P_{V}\right) \\ +f_{HP}\cdot f_{HD}\cdot f_{H\_S}\cdot f_{HT12}\left(f_{RHL}\left(D_{HL}+\eta\cdot D_{HL}\cdot D_{VL}\right)+\eta\cdot D_{RHL}\cdot f_{RVL}\cdot D_{VL}\right)p_{F4}\cdot s\left(1-P_{V}\right) \end{matrix}$$

Element *b* in matrix *M* is given by

$$b=R_{IHP\_2}+R_{IHD\_2}+R_{IHS\_2}+R_{IHT1\_2}+R_{IHT2\_2}+R_{IHL\_2}$$

whereby

$$\begin{matrix} R_{IHP\_2}=\left(D_{HP}\cdot p_{F1}+\eta\cdot D_{HP}\left(D_{VP}\cdot p_{F1}+f_{VP}\left(D_{VD}\cdot p_{F2}+f_{VD}\left(\begin{matrix} D_{VS}\cdot p_{F3}+f_{VS} \\ \left(\begin{matrix} D_{VT1}\cdot p_{F3}+f_{VT1} \\ \left(\begin{matrix} D_{VT2}\cdot p_{F3} \\ +f_{VT2}\cdot f_{RVL}\cdot D_{VL}\cdot p_{F4} \end{matrix}\right) \end{matrix}\right) \end{matrix}\right)\right)\right)\right)s\cdot P_{V} \\ +\eta\cdot D_{HP}\cdot f_{VP}\cdot f_{VD2}\cdot f_{RVL}\cdot D_{VL}\cdot p_{F4}\cdot s\cdot P_{V} \\ +\eta\cdot D_{HP}\cdot f_{VP}\cdot f_{VD}\cdot f_{VS}\cdot f_{VT12}\cdot f_{RVL}\cdot D_{VL}\cdot p_{F4}\cdot s\cdot P_{V} \end{matrix}$$

$$\begin{matrix} R_{IHD\_2}=f_{HP}\left(D_{HD}\cdot p_{F2}+\eta\cdot D_{HD}\left(D_{VD}\cdot p_{F2}+f_{VD}\left(D_{VS}\cdot p_{F3}+f_{VS}\left(\begin{matrix} D_{VT1}\cdot p_{F3}+f_{VT1} \\ \left(\begin{matrix} D_{VT2}\cdot p_{F3} \\ +f_{VT2}\cdot f_{RVL}\cdot D_{VL}\cdot p_{F4} \end{matrix}\right) \end{matrix}\right)\right)\right)\right)s\cdot P_{V} \\ +\eta\cdot D_{HD}\cdot f_{VD2}\cdot f_{RVL}\cdot D_{VL}\cdot p_{F4}\cdot s\cdot P_{V} \\ +\eta\cdot D_{HD}\cdot f_{VD}\cdot f_{VS}\cdot f_{VT12}\cdot f_{RVL}\cdot D_{VL}\cdot p_{F4}\cdot s\cdot P_{V} \end{matrix}$$

$$\begin{matrix} R_{IHS\_2}=f_{HP}\cdot f_{HD}\left(D_{HS}\cdot p_{F3}+\eta\cdot D_{HS}\left(D_{VS}\cdot p_{F3}+f_{VS}\left(D_{VT1}\cdot p_{F3}+f_{VT1}\left(\begin{matrix} D_{VT2}\cdot p_{F3} \\ +f_{VT2}\cdot f_{RVL}\cdot D_{VL}\cdot p_{F4} \end{matrix}\right)\right)\right)\right)s\cdot P_{V} \\ +\eta\cdot D_{HS}\cdot f_{VS}\cdot f_{VT12}\cdot f_{RVL}\cdot D_{VL}\cdot p_{F4}\cdot s\cdot P_{V} \end{matrix}$$

$$\begin{matrix} R_{IHT1\_2}=f_{HP}\cdot f_{HD}\cdot f_{H\_S}\left(D_{HT1}\cdot p_{F3}+\eta\cdot D_{HT1}\left(D_{VT1}\cdot p_{F3}+f_{VT1}\left(D_{VT2}\cdot p_{F3}+f_{VT2}\cdot f_{RVL}\cdot D_{VL}\cdot p_{F4}\right)\right)\right)s\cdot P_{V} \\ +\eta\cdot D_{HT1}\cdot f_{VT12}\cdot f_{RVL}\cdot D_{VL}\cdot p_{F4}\cdot s\cdot P_{V} \end{matrix}$$

$$R_{IHT2\_2}=f_{HP}\cdot f_{HD}\cdot f_{H\_S}\cdot f_{HT1}\left(D_{HT2}\cdot p_{F3}+\eta\cdot D_{HT2}\left(D_{VT2}\cdot p_{F3}+f_{VT2}\cdot f_{RVL}\cdot D_{VL}\cdot p_{F4}\right)\right)s\cdot P_{V}$$

$$\begin{matrix} R_{IHL\_2}=f_{HP}\cdot f_{HD}\cdot f_{H\_S}\cdot f_{HT1}\cdot f_{HT2}\left(f_{RHL}\left(D_{HL}+\eta\cdot D_{HL}\cdot D_{VL}\right)+\eta\cdot D_{RHL}\cdot f_{RVL}\cdot D_{VL}\right)p_{F4}\cdot s\cdot P_{V} \\ +f_{HP}\cdot f_{HD2}\left(f_{RHL}\left(D_{HL}+\eta\cdot D_{HL}\cdot D_{VL}\right)+\eta\cdot D_{RHL}\cdot f_{RVL}\cdot D_{VL}\right)p_{F4}\cdot s\cdot P_{V} \\ +f_{HP}\cdot f_{HD}\cdot f_{H\_S}\cdot f_{HT12}\left(f_{RHL}\left(D_{HL}+\eta\cdot D_{HL}\cdot D_{VL}\right)+\eta\cdot D_{RHL}\cdot f_{RVL}\cdot D_{VL}\right)p_{F4}\cdot s\cdot P_{V} \end{matrix}$$

Element *c* in matrix *M* is given by

$$c=R_{IVP\_1}+R_{IVD\_1}+R_{IVS\_1}+R_{IVT1\_1}+R_{IVT2\_1}+R_{IVL\_1}$$

whereby

$$\begin{matrix} R_{IVP\_1}=D_{VP}\cdot p_{F1}\cdot s\cdot\left(1-P_{V}\right) \\ R_{IVD\_1}=f_{VP}\cdot D_{VD}\cdot p_{F2}\cdot s\cdot\left(1-P_{V}\right) \\ R_{IVS\_1}=f_{VP}\cdot f_{VD}\cdot D_{VS}\cdot p_{F3}\cdot s\cdot\left(1-P_{V}\right) \\ R_{IVT1\_1}=f_{VP}\cdot f_{VD}\cdot f_{VS}\cdot D_{VT1}\cdot p_{F3}\cdot s\cdot\left(1-P_{V}\right) \\ R_{IVT2\_1}=f_{VP}\cdot f_{VD}\cdot f_{VS}\cdot f_{VT1}\cdot D_{VT2}\cdot p_{F3}\cdot s\cdot\left(1-P_{V}\right) \\ R_{IVL\_1}=f_{VP}\left(\begin{matrix} f_{VD}\cdot f_{VS}\cdot f_{VT1}\cdot f_{VT2}\cdot f_{RVL}\cdot D_{VL}+f_{VD2}\cdot f_{RVL}\cdot D_{VL} \\ +f_{VD}\cdot f_{VS}\cdot f_{VT12}\cdot f_{RVL}\cdot D_{VL}+f_{VD2}\cdot f_{RVL}\cdot D_{VL} \end{matrix}\right)\cdot p_{F4}\cdot s\cdot\left(1-P_{V}\right) \end{matrix}$$

Element *d* in matrix *M* is given by

$$d=R_{IVP\_2}+R_{IVD\_2}+R_{IVS\_2}+R_{IVT1\_2}+R_{IVT2\_2}+R_{IVL\_2}$$

whereby

$$\begin{matrix} R_{IVP\_2}=D_{VP}\cdot p_{F1}\cdot s\cdot P_{V} \\ R_{IVD\_2}=f_{VP}\cdot D_{VD}\cdot p_{F2}\cdot s\cdot P_{V} \\ R_{IVS\_2}=f_{VP}\cdot f_{VD}\cdot D_{VS}\cdot p_{F3}\cdot s\cdot P_{V} \\ R_{IVT1\_2}=f_{VP}\cdot f_{VD}\cdot f_{VS}\cdot D_{VT1}\cdot p_{F3}\cdot s\cdot P_{V} \\ R_{IVT2\_2}=f_{VP}\cdot f_{VD}\cdot f_{VS}\cdot f_{VT1}\cdot D_{VT2}\cdot p_{F3}\cdot s\cdot P_{V} \\ R_{IVL\_2}=f_{VP}\left(\begin{matrix} f_{VD}\cdot f_{VS}\cdot f_{VT1}\cdot f_{VT2}\cdot f_{RVL}\cdot D_{VL}+f_{VD2}\cdot f_{RVL}\cdot D_{VL} \\ +f_{VD}\cdot f_{VS}\cdot f_{VT12}\cdot f_{RVL}\cdot D_{VL}+f_{VD2}\cdot f_{RVL}\cdot D_{VL} \end{matrix}\right)\cdot p_{F4}\cdot s\cdot P_{V} \end{matrix}$$

For each of the 4 elements of *M* the *R*'s denote the contribution of the 6 infectious compartments *I_HP_*, *I_HD_*, *I_HS_*, *I_HT1_*, *I_HT2_* and *I_HL_* (humans without HIV) and *I_VP_*, *I_VD_*, *I_VS_*, *I_VT1_*, *I_VT2_* and *I_HL_* (humans with HIV) to *R_0_*, the basic reproduction number. *R_0_* is the largest eigenvalue of the next generation matrix *M*, whereby eigenvalues *λ_1,2_* are given by
